# Endothelial barrier and sepsis: mechanisms and potential therapeutic strategies

**DOI:** 10.1016/j.mmr.2026.100013

**Published:** 2026-04-04

**Authors:** Yan-Hong Jiang, Xing-Juan Li, De-Cao Ma, Yong-Lin Chen, Yi-Yu Shi, Yang Lu, Ren-Fang Mao

**Affiliations:** a Department of Pathophysiology, School of Medicine, Nantong University, Nantong 226001, Jiangsu, China; b Department of Critical Care, Affiliated Hospital of Nantong University, Nantong 226001, Jiangsu, China; c Department of Pathology, the First Hospital of Lanzhou University, Lanzhou 730000, China

**Keywords:** Sepsis, Endothelial dysfunction, Vascular permeability, Glycocalyx injury, Therapeutic strategies

## Abstract

Sepsis is a life-threatening condition characterized by an exaggerated and uncontrolled immune response, leading to widespread inflammation throughout the body. This immune response can compromise the integrity of the endothelial barrier, resulting in increased vascular permeability. The degree of vascular permeability in septic shock patients correlates with disease severity and significantly influences the outcomes of resuscitation and prognosis. This review systematically examines the structural regulation of the endothelial barrier and the dynamic mechanisms of its injury in subgroups of sepsis. In response to these mechanisms, emerging therapeutic strategies focus on glycocalyx protection, signal pathway modulation, cytoskeleton stability, and immune regulation, aiming to restore endothelial barrier function through multi-target synergism. In the future, combining analysis of endothelial barrier function and the dynamic regulation mechanism provides a new perspective for the precise treatment of sepsis.

## 1 Background

Sepsis is characterized by life-threatening organ dysfunction due to a dysregulated host response to infection [1]. It is a leading cause of morbidity and mortality worldwide, particularly among vulnerable populations such as neonates and pregnant women [2]. According to the Global Burden of Disease analysis, neonatal and maternal sepsis were responsible for an estimated 223,200 neonatal deaths and 26,700 maternal deaths globally in 2023 [2]. For many survivors, there remains an ongoing risk of death following hospital discharge, as well as long-term cognitive and functional deficits [3].

The pathogenesis of sepsis-induced endothelial dysfunction is complex and multifactorial [4], [5]. Growing evidence suggests that the underlying mechanisms may vary considerably across different patient subgroups, influenced by factors such as the nature of the pathogen, host age, and the temporal stage of the disease [5], [6], [7]. Despite this heterogeneity, a central pathophysiological feature is the dysregulation of vascular permeability. Increased vascular permeability represents a key driver in sepsis, leading to the abnormal extravasation of vascular fluid into surrounding tissues [8]. Clinically, sepsis is often associated with progressive subcutaneous and body cavity edema, a form of tissue swelling resulting from exudation [9], [10]. Vascular leakage and tissue edema in sepsis are indicative of endothelial dysfunction [11], significantly contributing to multiple organ dysfunction and elevated mortality rates. The endothelial cell (EC) barrier is critical for the regulation of nutrient exchange between the blood and surrounding tissues. Under normal conditions, the endothelium forms a selective layer that regulates the exchange of fluids and solutes with surrounding tissues [12]. However, during sepsis, the integrity of intercellular contacts in postcapillary venules is compromised, leading to the formation of intercellular spaces. The resulting edema can precipitate shock and multiorgan failure [13], [14]. Therefore, endothelial dysfunction is central to the pathophysiology of sepsis [15].

This review provides a comprehensive analysis of the molecular mechanisms underlying sepsis-induced endothelial dysfunction and explores potential therapeutic strategies targeting these mechanisms. The aim is to deepen the understanding of the vascular pathophysiology of sepsis and provide insights for the development of new clinical interventions.

## 2 Structure and function of the endothelial barrier and its regulation

The inner surface of blood vessels is lined with a monolayer of tightly organized ECs, forming a continuous vascular endothelium through specialized junctions [16]. This structural layer serves not only as a physical barrier to preserve vascular integrity and maintain blood volume, but also contributes actively to various physiological regulatory processes, including blood pressure regulation [17], anti-coagulation [18], and pro-coagulation [19]. The regulation of blood volume is one of the primary physiological characteristics of the endothelium. It is achieved by fluid exchange across the microvascular endothelium, which is governed by hydrostatic and oncotic pressure gradients, as well as the integrity and functional properties of the endothelial barrier. Regulating and maintaining blood pressure is another pivotal physiological function of the endothelium. ECs inhibit platelet aggregation by synthesizing and releasing endothelial nitric oxide (NO) synthase (eNOS) and prostacyclin (also known as prostaglandin I_2_), secrete endothelin-1, and promote angiotensin II production to precisely regulate vasoconstriction and tension [20]. In terms of coagulation, the thrombomodulin expressed on the surface of ECs binds to thrombin, activating the protein C system and serving an essential anti-coagulant role [21]. Under normal physiological conditions, ECs maintain the dynamic balance of the coagulation-fibrinolysis system by regulating the expression and activity of pro-coagulant factors [such as tissue factor (TF) and von Willebrand factor (vWF)] and anti-coagulant/fibrinolytic factors (such as thrombomodulin and tissue plasminogen activator) to prevent hemorrhagic or thrombotic events [22]. However, during severe sepsis, persistent inflammation drives the hemostatic system toward a hypercoagulable and hypofibrinolytic state, resulting in widespread microvascular thrombosis, ischemic organ damage, and eventual progression to multiple organ dysfunction [22], which will be further elaborated upon in the subsequent sections. In addition, the endothelium is now recognized as a highly dynamic interface that not only functions as a metabolic interface with considerable diversity across various organs but also communicates instructive signals to regulate organ function in an angiocrine manner. Furthermore, the endothelium is essential for cell trafficking and perfusion, and critically regulates vascular permeability and barrier function [23].

**Fig. 1:**
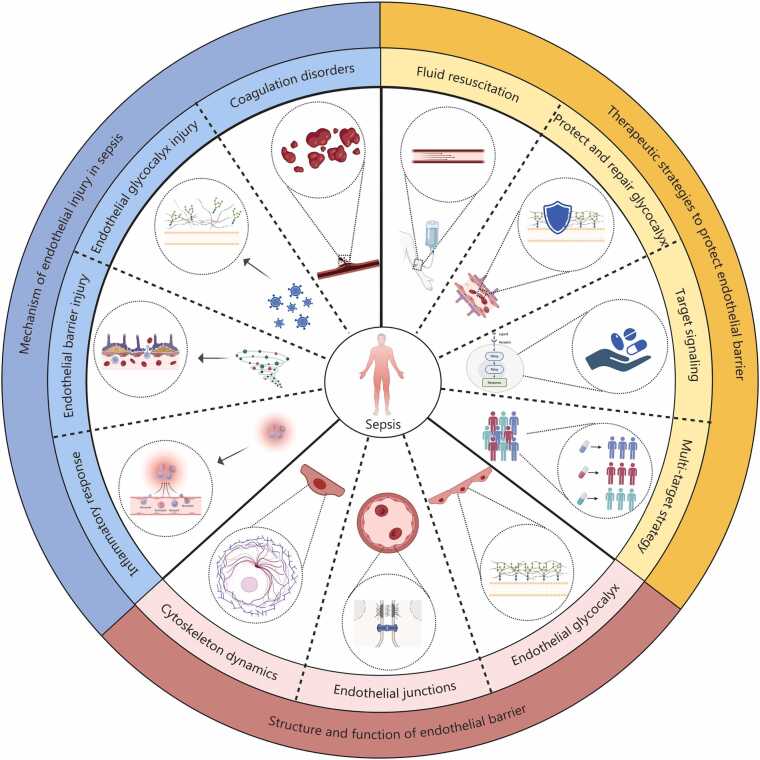
Schematic overview of the “mechanism-structure-strategy” framework in sepsis-induced endothelial barrier injury. This diagram summarizes the core pathophysiological cascade of endothelial injury in sepsis and outlines corresponding therapeutic rationale. The bottom panel depicts the structural basis of the endothelial barrier, emphasizing endothelial glycocalyx, intercellular junctions, and cytoskeletal dynamics as the ultimate targets of damage. The left panel illustrates key injury mechanisms, including inflammation response, endothelial dysfunction, endothelial barrier injury, and coagulation disorders, which collectively contribute to organ hypoperfusion. The right panel outlines potential multi-target therapeutic strategies of glycocalyx protecting and repairing, target signaling modulation, fluid resuscitation, and multi-target strategy to restore endothelial integrity. Created with BioRender.com.

The endothelial barrier, which separates blood from tissue, is composed of the glycocalyx on the vascular endothelium, the ECs themselves, and their intercellular junctions [24] (Fig. 1**, bottom**). The functional characteristics of this barrier vary significantly across organs, as the properties of the ECs differ considerably between tissues [25], [26]. For example, brain microvascular ECs form a highly selective blood-brain barrier through tight junctions (TJs), which effectively blocks the transcellular transport of plasma proteins and most small molecules, thereby protecting neural tissue [27]. On the contrary, the glomerular capillary endothelium features fenestrated pores with diameters ranging from 60 to 80 nm, making it nearly completely permeable to water and low-molecular-weight solutes, thereby facilitating efficient filtration [25]. Sepsis can provoke a highly heterogeneous endothelial response, manifesting as characteristic damage in different organs: the kidney is marked by increased microvascular permeability and impaired perfusion, the lung is affected by pulmonary edema due to barrier function disruption, and the liver is characterized by sinusoidal collapse and immune filtration dysfunction [22]. This organ-specific variation in endothelial response is a key factor underlying the diversity and severity of organ dysfunction observed in sepsis.

### 2.1 Endothelial glycocalyx (eGC)

The eGC is a gel-like layer approximately 0.2−0.5 μm in thickness that covers the vascular endothelium. It is primarily composed of glycosaminoglycans and proteoglycans, which are rich in heparan sulfate (HS), and is anchored to the EC surface via glycosaminoglycans [28]. Proteoglycans are the predominant components of the glycocalyx and are covalently linked to glycosaminoglycans, forming soluble proteoglycans. The soluble component exists in a dynamic equilibrium, a process that continuously influences the thickness of the glycocalyx [29]. The high density of sulfate and sialic acid groups imparts a strong negative charge, which creates both size- and charge-selective hindrance, sustaining a low sub-glycocalyx oncotic pressure. This mechanism contributes to the revised Starling principle and limits the entry of albumin-rich fluid [30]. The revised Starling principle underscores the glycocalyx and subglycocalyx oncotic pressure as key determinants of net filtration [30]. Steady-state reabsorption across continuous capillaries is limited, and lymphatics return filtered fluid [31]. The endothelial surface layer includes the glycocalyx, along with loosely associated plasma proteins (e.g., albumin, orosomucoid), which together form a dynamic, adsorbed layer [32]. Albumin normally traverses the endothelium predominantly via caveolae-mediated transcytosis and is partially retained by the glycocalyx [33]. During sepsis, degradation of the glycocalyx and opening of intercellular junctions increase the leakage of albumin into the interstitium, thereby altering colloid osmotic forces and promoting edema [34]. Inflammatory mediators activate enzymatic systems that rapidly degrade the eGC, releasing it into blood/urine, resulting in a thinner glycocalyx layer with a sparser structure [35]. Following the loss of barrier function, vascular permeability increases, leading to tissue edema, microthrombosis, leukocyte adhesion, and abnormal vasodilation. These changes cause microcirculatory disturbances and organ failure, and the concentration of glycocalyx fragments in blood and urine is positively correlated with disease severity and the risk of mortality [36].

### 2.2 Endothelial cells to cell junctions

**Fig. 2:**
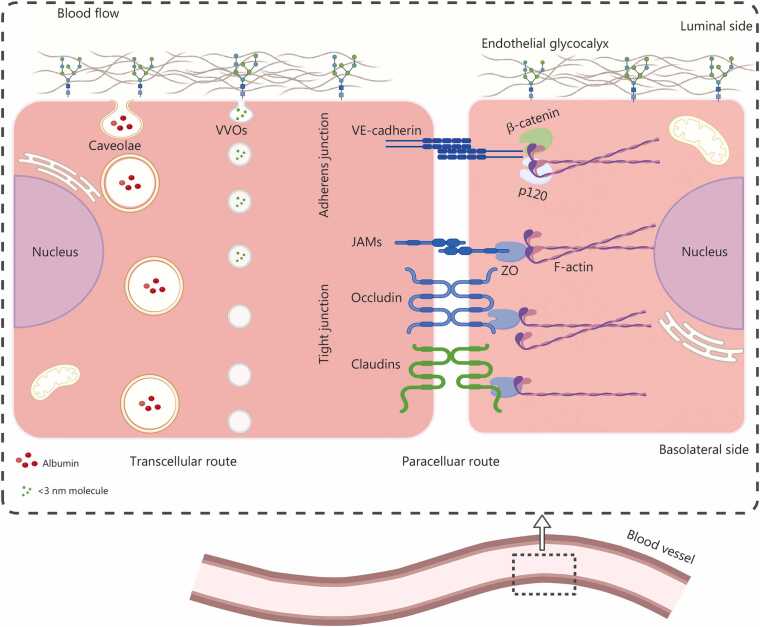
Structure and transport pathways of the endothelial barrier. This schematic illustrates the endothelial cell layer as a selective barrier, outlining 2 principal routes for substance exchange across a vascular cross-section. The transcellular pathway (left) mediates transport of larger molecules (e.g., albumin) through the endothelial cytoplasm via caveolae and the vesiculo-vacuolar organelle (VVO) system, involving endocytosis, intracellular trafficking, and exocytosis. The paracellular pathway (right) permits passive diffusion of small solutes and ions through the intercellular space, a process dynamically controlled by endothelial junctional complexes. These include tight junctions [composed of transmembrane proteins such as occludin, claudins, and junctional adhesion molecules (JAMs) connected to the actin cytoskeleton through scaffold proteins zonula occludens (ZO), which are essential for barrier integrity], adherens junctions [composed of vascular endothelial (VE)-cadherin and associated intracellular adaptors (e.g., β-catenin, p120), which provide mechanical stability]. The luminal endothelial glycocalyx serves as an initial molecular sieve. Cytoskeletal dynamics, mediated by ZO proteins linking junctions to filamentous actin (F-actin), are critical for maintaining barrier function and regulating permeability. Created with BioRender.com.

The endothelial barrier dynamically regulates the transendothelial passage of fluids, solutes, plasma proteins, and leukocytes by precisely controlling the opening and closing of intercellular junctions [12]. Current evidence suggests that substance transport primarily occurs through two pathways: transcytosis across ECs and paracellular transport via transient openings in the intercellular junctions [37] (Fig. 2). The paracellular pathway, also referred to as interendothelial junctions (IEJs), is maintained through endothelial-to-endothelial contacts between adjacent cells and the surface glycocalyx layer. IEJs include TJs, adherens junctions (AJs), and gap junctions composed of connexins [38]. The actin cytoskeleton is closely associated with each junction and regulates its integrity through actin remodeling [39].

### 2.3 Tight junction (TJs)

TJs comprise approximately 1/4 of all endothelial junctions and are essential for maintaining the vascular endothelial barrier. TJs are formed by claudins, occludins, and adhesion molecules, which interact with cytosolic scaffold proteins such as zonula occludens (ZO)-1, ZO-2, and ZO-3 [40]. ZO-1 is the skeleton anchor point of TJs, directly bridging transmembrane proteins and actin to better regulate TJ function [41]. Occludin, the first TJ protein identified in ECs, is expressed in both endothelial and epithelial cells. It regulates the formation and breakdown of TJs and controls the activity of transmembrane proteins (such as actin and myosin) to preserve TJ integrity [42].

### 2.4 Adherens junctions (AJs)

AJs govern endothelial permeability in venous circulation [43]. AJs are mediated by cadherins, with vascular endothelial cadherin (VE-cadherin), also known as CDH5, being the key component of these junctions [44]. VE-cadherin is expressed not only in vascular ECs but also in lymphatic ECs and certain hematopoietic cells [45]. Its structure consists of extracellular cadherin motifs, transmembrane domains, and intracellular domains, all of which are essential for maintaining vascular integrity [12]. In sepsis, metalloproteinases induce the proteolysis of endothelial cadherins [46]. And cytokines promote the internalization of VE-cadherin into a soluble form that can be detected in plasma, thereby promoting vascular permeability [47]. Permeability-increasing agents also stimulate calcium influx and induce tyrosine phosphorylation of VE-cadherin and β-catenin, leading to the weakening or disruption of endothelial AJs [48]. Additionally, the angiopoietin (Ang)/Tie signaling axis is a critical regulator of vascular homeostasis. Studies have found that *Ang2* deficiency can reduce vascular leakage caused by sepsis [49], [50], [51], [52]. Therefore, Ang2 may have detrimental effects in sepsis. Further study found that the interaction between Tie1 and Tie2 is destroyed, resulting in the loss of Ang2’s function of activating Tie2, which in turn exerts an antagonistic effect, thereby weakening the stability of the vascular barrier and promoting vascular leakage in sepsis [53].

### 2.5 Cytoskeletal dynamics

The endothelial cytoskeleton is critical to the formation and maintenance of the endothelial barrier, as well as in regulating permeability. It is composed of 3 interconnected networks: actin microfilaments, intermediate filaments, and microtubules. Actomyosin contraction and increased cytoskeletal tension are key mechanisms that contribute to endothelial hyperpermeability [54]. The dynamics of the actin cytoskeleton also regulate endothelial permeability. For example, the small GTPase Ras homolog gene family member A (RhoA) stimulates the formation of actin stress fibers and promotes EC contraction. This results in changes in the shape of ECs, disassembly of the cortical actin rims, and redistribution of actin into cytoplasmic stress fibers, ultimately increasing vascular permeability [55]. Additionally, sphingosine-1-phosphate (S1P), an important bioactive lipid, significantly contributes to maintaining the restrictive function of the endothelial barrier and counteracting the effects of permeability-increasing agents [56]. S1P, predominantly carried by high-density lipoprotein and albumin, supports barrier integrity via sphingosine-1-phosphate receptor 1-inhibitory G protein-Ras-related C3 botulinum toxin substrate (S1PR1-Gi-Rac) signaling, promoting the stabilization of cortical actin and tight VE-cadherin junctions [56], [57]. Counteracting the G12/13-RhoA pathways can induce contraction and promote leak [58]. Platelet-released S1P acutely enhances transendothelial resistance, closes intercellular gaps, and mitigates edema [59].

## 3 Mechanism of endothelial injury in sepsis

### 3.1 Dysregulated coagulation

Under physiological conditions, ECs form a non-adherent surface that prevents platelet activation and the initiation of coagulation cascades [60]. TF is a pro-coagulant transmembrane glycoprotein synthesized by ECs and white blood cells [61]. When forming a complex with factor VIIa, factors IX and X are activated to promote clot formation; ECs bind to factor Xa by producing tissue factor pathway inhibitor (TFPI), inhibiting TF-factor VIIa complex and limiting fibrin deposition, while thrombomodulin and endothelial protein C receptor activator protein C inhibits factor V, factor VIII, and plasminogen activator inhibitor-1 (PAI-1) to regulate anticoagulation [22]. In the blood circulation, the large polymer of vWF is rapidly processed into smaller, less active complexes by the metalloprotease ADAMTS-13 (also known as vWF lyase, containing the thrombospondin type 1 repeat sequence) [22].

In sepsis, interleukin (IL)-6 and plasma-free hemoglobin directly inactivate ADAMTS-13 [62]. Activated neutrophil-derived reactive oxygen species (ROS) further inhibit ADAMTS-13 activity [63]. The absence of ADAMTS-13 prevents the cleavage of unusually large vWF, thereby increasing the risk of thrombosis [64]. During sepsis, EC-associated anti-coagulation and fibrinolysis are also severely impaired [65], [66]. These disruptions lead to the release of PAI-1 from ECs and monocytes. Elevated plasma PAI-1 increases the risk of thrombosis and is associated with multiple organ dysfunction and mortality in patients [67]. Notably, there is a two-way positive feedback between inflammation and coagulation: inflammation initiates and spreads coagulation, which in turn significantly aggravates inflammation [68]. This persistent cycle directly promotes the formation of microvascular thrombi, which are composed of fibrin, activated platelets, and entrapped leukocytes [69].

It is important to note that microvascular immune thrombosis is a central mechanism driving coagulopathy, organ hypoperfusion, and death. Platelets and red blood cells are the 2 major promoters of this process. The interaction between platelets and white blood cells aggravates the inflammatory response and hinders microvascular blood flow [70], [71]. Red blood cells are spherical, and the reduction of negative charge on the surface leads to the aggregation of red blood cells, resulting in increased thrombosis [72]. At the same time, the decrease in erythrocyte deformability is also closely associated with increased mortality in sepsis patients [73].

### 3.2 Endothelial barrier dysfunction

Although the core elements of endothelial dysfunction in sepsis are well-recognized, its mechanisms are likely to vary across patient subgroups (e.g., bacterial vs. viral sepsis, young vs. elderly cohorts, early vs. late disease stages). A more comprehensive understanding of this heterogeneity is therefore crucial for advancing beyond a one-size-fits-all treatment approach. Sepsis-induced endothelial barrier dysfunction is triggered by systemic inflammation, ultimately leading to the disintegration and cytoskeleton remodeling [22], [74], but it is worthy of note that the endothelial inflammatory responses to infection are evolutionarily conserved and serve critical normal survival functions.

**Fig. 3:**
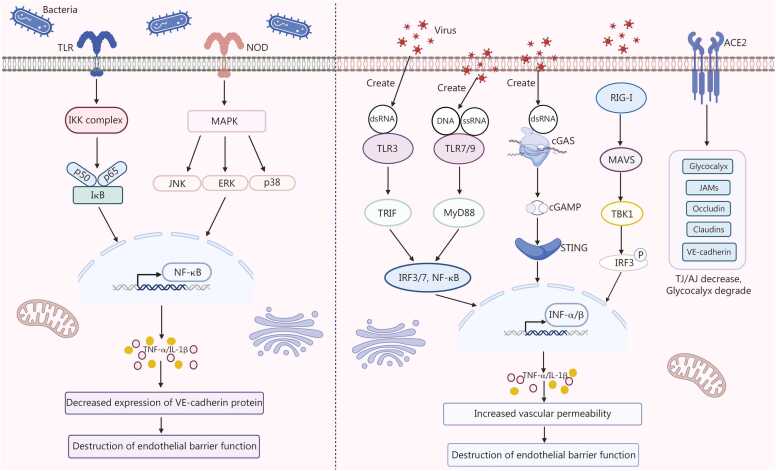
Molecular mechanisms of endothelial barrier disruption in bacterial vs. viral sepsis. This diagram compares the distinct upstream signaling pathways triggered by bacterial (left) and viral (right) infections, which converge to disrupt endothelial barrier integrity. Bacterial pathway (left): bacterial pathogen-associated molecular patterns, such as lipopolysaccharide (LPS), are recognized by Toll-like receptors (TLR) (e.g., TLR4) and NOD-like receptors (e.g., NOD1/2) on endothelial cells. Signaling through these receptors activates NF-κB (via IKK complex) and MAPK pathways (JNK, ERK, p38), driving the production of pro-inflammatory cytokines (e.g., TNF-α, IL-1β). These cytokines directly downregulate the adherens junction protein VE-cadherin, weakening the endothelial barrier. Viral pathway (right): viral nucleic acids (dsRNA, ssRNA, DNA) are detected by endosomal TLR3/7/9 or cytosolic sensors (e.g., cGAS). These signals activate transcription factors IRF3/7 and NF-κB, inducing the production of type I interferons (IFN-α/β) and inflammatory cytokines. IFN-α/β may upregulate viral receptors (e.g., ACE2). The resulting cytokine storm downregulates tight-junction proteins (occludin, claudins, JAMs), reduces VE-cadherin expression, and degrades the luminal glycocalyx, collectively exacerbating vascular leakage. Although bacterial and viral sepsis engage different pattern-recognition receptors, their downstream effects converge on the activation of shared transcription factors and a pronounced inflammatory response, ultimately targeting endothelial junctional complexes and the glycocalyx to compromise barrier function. Created with BioRender.com. NOD. Nucleotide-binding oligomerization domain; NF-κB. Nuclear factor kappa-light-chain-enhancer of activated B cells; IKK. IκB kinase; MAPK. Mitogen-activated protein kinase; JNK. C-Jun N-terminal kinase; ERK. Extracellular signal-regulated kinase; TNF-α. Tumor necrosis factor-α; IL-1β. Interleukin-1 beta; VE-cadherin. Vascular endothelial-cadherin; dsRNA. Double-stranded RNA; ssRNA. Single-stranded RNA; DNA. Deoxyribonucleic acid; cGAS. Cyclic guanosine monophosphate-adenosine monophosphate synthase; IRF3/7. Interferon regulatory factor 3/7; IFN-α/β. Interferon-α/β; ACE2. Angiotensin-converting enzyme 2; JAMs. Junctional adhesion molecules; PRRs. pattern-recognition receptors; MAVS. Mitochondrial antiviral-signaling protein; TBK1. Tank-binding kinase 1; cGAMP. cyclic Guanosine monophosphate-adenosine monophosphate; TRIF. Tir-domain-containing adapter-inducing interferon-β; STING. Stimulator of interferon genes; TJ. Tight Junction; AJ. Adherens junction; JAMs. Junctional adhesion molecules.

Since sepsis is caused by the host’s immune imbalance in response to infection, the destruction of the endothelial barrier in sepsis also follows a typical inflammation-driven pattern (Fig. 3). The initial event involves the recognition of pathogen-associated molecular patterns and damage-associated molecular patterns, which trigger a massive release of pro-inflammatory cytokines, including tumor necrosis factor-α (TNF-α) and IL-1β [75], [76]. These cytokines bind to their receptors on ECs, activating intracellular signaling pathways such as nuclear factor kappa B (NF-κB) and p38 mitogen-activated protein kinase (MAPK) [77]. This activation induces a pro-inflammatory and pro-adhesive endothelial phenotype, characterized by increased expression of adhesion molecules like intercellular adhesion molecule-1 (ICAM-1) and further production of ROS, creating a self-perpetuating cycle of amplification [19]. The convergence of these signals leads to 2 pivotal structural alterations: 1) the disintegration of AJs, primarily through the internalization, phosphorylation, and enzymatic cleavage of the key adhesive protein VE-cadherin [78]; and 2) actin-myosin-mediated cell contraction, instigated by the phosphorylation of myosin light chain (MLC) [79]. Concurrently, the protective glycocalyx layer is shed from the endothelial surface [80]. As a normal physiological response to localized infection, AJs disruption and cell contraction facilitate leukocyte transmigration and tissue swelling. However, during sepsis, wide-spread systemic activation of these mechanisms leads to massive, uncontrolled fluid transmigation into the interstitium and driving tissue edema: a hallmark of organ dysfunction in sepsis.

#### 3.2.1 Regulation of endothelial injury mechanisms in bacterial and viral sepsis

The nature of the invading pathogen critically influences the endothelial response. Bacterial sepsis and viral sepsis differ significantly in pathogen recognition, immune response activation, and clinical pathological manifestations. Bacterial sepsis is primarily triggered by cell wall components of Gram-positive bacteria (e.g., *Staphylococcus aureus*, *Streptococcus*) and Gram-negative bacteria (e.g., *Escherichia coli*), such as lipopolysaccharide (LPS), peptidoglycan, and lipoteichoic acid, which activate the host immune system via Toll-like receptors (TLR2, TLR4, TLR5, TLR9) and NOD-like receptors (NOD1, NOD2) [81], [82]. LPS, the core molecule responsible for endothelial barrier dysfunction, binds to TLR4 on the surface of ECs and activates robust intracellular signaling pathways [83]. It disrupts endothelial barrier function through multiple molecular pathways. On one hand, LPS induces the phosphorylation and subsequent internalization of VE-cadherin from the plasma membrane [84]. Internalized VE-cadherin, which associates with the small GTPase Rab7, is trafficked to lysosomes for degradation, leading to a decrease in total VE-cadherin protein expression and, consequently, a persistent loss of endothelial barrier function [85]. On the other hand, LPS activates the RhoA/Rho-associated coiled-coil kinase (ROCK) and myosin light-chain kinase (MLCK) pathways, resulting in phosphorylation of MLC [86]. This triggers actin-myosin contraction, causing EC retraction and widening of intercellular gaps. Furthermore, LPS activates transient receptor potential canonical calcium channels, elevating intracellular calcium concentrations, which in turn activate calmodulin and MLCK, exacerbating cellular contraction [39]. Additionally, through an indirect mechanism, circulating LPS forms complexes with lipoproteins such as chylomicrons. These complexes traverse the endothelial barrier via scavenger receptor (SR-BI/CD36)-mediated transcytosis [39]. Subsequently, LPS polarizes tissue-resident macrophages towards the M1 phenotype. Activated M1 macrophages release large quantities of pro-inflammatory cytokines, including TNF-α and IL-6, which perpetually assault ECs from the abluminal side, amplifying the disruption of intercellular junctions [87]. Notably, Src kinase-mediated tyrosine phosphorylation of VE-cadherin (e.g., Y658, Y731) is a central event in the destruction of AJs, leading to the dissociation of VE-cadherin from catenin and the disintegration of the junction structure [88].

In contrast, viral sepsis [e.g., influenza, herpes simplex virus, severe acute respiratory syndrome coronavirus 2 (SARS-CoV-2)] is primarily initiated by viral nucleic acids (single-stranded RNA, double-stranded RNA, DNA) recognized by receptors such as TLR3, TLR7, TLR9, retinoic acid-inducible gene I (RIG-I), triggering an antiviral immune response dominated by type I interferons (interferon-α/β) [89], [90]. This response is often accompanied by lymphocyte activation, endotheliitis, and complement activation, which are frequently observed in respiratory viral infections or immunocompromised patients [91], [92]. Unlike bacterial sepsis, which primarily activates systemic inflammatory responses through endotoxin (such as LPS), viral sepsis often causes more direct and specific damage to the endothelial barrier [89]. Many viruses (such as dengue virus, hantavirus, SARS-CoV-2) can directly infect ECs by utilizing their surface-specific receptors (such as angiotensin-converting enzyme 2 into the cells), replicate, and lead to apoptosis, necrosis, or pyroptosis [93], [94]. This direct cytotoxic effect is infrequent in bacterial sepsis. Virus-encoded proteins (such as dengue virus NS1 protein, SARS-CoV-2 Spike protein) directly disrupt the connection structure between ECs, including the down-regulation of TJ proteins (occludin, claudin) and AJ protein (VE-cadherin), resulting in increased intercellular space and significantly elevated vascular permeability [95], [96]. Viral proteins (such as nonstructural protein) degrade the glycocalyx layer on the surface of ECs, and this destruction exacerbates fluid extravasation and tissue edema [97]. Besides, virus-activated immune responses (such as cytokine storm, NETosis, and complement activation) can indirectly damage ECs [98]. However, the initiation mechanism differs from that of bacterial sepsis. Viruses are more likely to trigger interferon response and inflammatory factor release through intracellular recognition pathways such as RIG-I and cyclic guanosine monophosphate-adenosine monophosphate synthase-stimulator of interferon genes, subsequently recruiting immune cells to attack the endothelial structure [89]. Therefore, although the causes of the 2 types of sepsis differ, they both ultimately cause significant damage to the endothelial barrier, indicating the need for distinct treatment strategies targeting each form.

#### 3.2.2 Regulation of age on the mechanism of endothelial injury

The aging endothelium exists in a pre-activated, fragile state, and its response to sepsis strikes has undergone fundamental changes compared to younger hosts. In elderly individuals, the endothelium is already in a state of dysfunction due to “inflammaging” and cellular senescence before encountering sepsis [99]. First of all, a chronic, low-grade systemic inflammatory state is its core feature. The baseline level of pro-inflammatory cytokines (such as IL-6) continued to rise, leaving ECs in a “pre-activated” primed state [100]. This means that when sepsis strikes, even a relatively mild infection stimulus is sufficient to trigger severe dysfunction and quickly break through the pathological threshold. Chronic diseases such as aging, hypertension, and diabetes contribute to the thinning and shedding of the protective eGC layer [101]. This not only destroys the first physical barrier to prevent liquid and protein leakage, but also exposes the following adhesion molecules, creating conditions for leukocyte adhesion [99]. Secondly, increased activity of nicotinamide adenine dinucleotide phosphate oxidase and uncoupled eNOS in senescent ECs leads to excessive production of ROS [102]. This oxidative environment can damage cellular proteins, lipids, and DNA, further exacerbating inflammation and dysfunction [103]. Chronic inflammation and oxidative stress lead to the degradation of VE-cadherin and other junction proteins, resulting in gaps between ECs and the destruction of blood-tissue barrier integrity [104]. Aging ECs show reduced eNOS activity and diminished bioavailability of NO, which impairs their anticoagulant function [105]. Therefore, the effects of sepsis and aging on endothelial function present a complex and organ-specific mechanism.

Clinical studies have indicated that EC activation markers in the systemic circulation of elderly patients with sepsis are low, suggesting that their systemic endothelial barrier dysfunction is relatively mild, which may be due to aging-related chronic inflammation-induced “EC tolerance” [106], [107], [108]. However, this systemic “passivation” response does not protect the microvascular system of specific organs. Animal models reveal that in the brain, sepsis in the elderly causes microvascular endothelial dysfunction, which is manifested by a decrease in NO-mediated vasodilation and damage to neurovascular coupling, which occurs before cognitive impairment [109]. More importantly, sepsis has been shown to directly induce p21-mediated acute cellular senescence in various cell types (including sinusoidal ECs) in organs such as the liver [110]. These aging ECs aggravate local inflammation, microcirculation disorders, and organ damage by secreting senescence-associated secretory phenotype factors [111], [112]. In summary, aging may slow down the response of systemic endothelium to sepsis stimulation, but at the same time aggravate the specific damage of sepsis to the microvascular endothelium of the brain, liver, and other organs, which highlights the importance of age and organ specificity in treatment strategies. Conversely, in younger patients, particularly neonates and infants, the hemostatic and endothelial systems are still developing and function in a manner distinct from adults [113]. In contrast to adults, neonates exhibit developmental hemostasis, characterized by reduced levels of vitamin K-dependent factors, natural anti-coagulants, and altered fibrinolytic activity. Their platelets are hypo-reactive, with reduced expression of key receptors such as protease-activated receptor (PAR-1 and PAR-4), and impaired degranulation and aggregation responses [114], [115]. Despite these limitations, neonates maintain hemostatic balance through compensatory mechanisms, including elevated hematocrit, increased vWF, and the presence of ultra-large multimers [116]. In the context of sepsis, neonates mount a tolerogenic and anti-inflammatory immune response, with attenuated cytokine production (e.g., TNF-α, IL-6) and reduced endothelial activation, which may mitigate initial inflammatory damage [117]. While they may not exhibit the hyper-inflammatory response seen in adults, their immature coagulation and immune systems render them highly susceptible to both thrombotic and hemorrhagic complications during sepsis [118]. However, neonatal ECs exhibit developmental-stage-specific responses, such as the massive production of IL-11, which may be involved in unique systemic inflammatory processes through hematopoiesis and immune regulation [119]. In neonatal sepsis, the core mechanism of endothelial barrier dysfunction begins with the severe shedding of the glycocalyx, and the level of its degradation product, syndecan-1 (SDC1), is directly related to mortality [120]. Mechanistically, glycocalyx hydrolase (heparinase III) triggers SDC1 shedding, which subsequently activates the NF-κB pathway, resulting in down-regulation of TJ protein ZO-1, and ultimately increased vascular permeability [120]. At the epigenetic level, sepsis stimulation (such as LPS) activates the self-perpetuating feedback loop of transcription factor forkhead box protein C2 by inducing histone H3 lysine 27 acetylation [121]. This mechanism may be particularly active in developing ECs, driving the abnormal transformation of cell identity to a lymphoid phenotype, and exacerbating pulmonary vascular remodeling and dysfunction. Consequently, age is not merely a demographic variable but a fundamental biological determinant of endothelial resilience during sepsis.

#### 3.2.3 Endothelial dysfunction in early and late sepsis

Sepsis is a dynamic process, and the mechanisms controlling endothelial dysfunction evolve significantly from the initial hours of the disease to the continuous stage [77], [122]. In the early hyperdynamic period, endothelial dysfunction is primarily driven by severe inflammatory signals such as TNF-α, IL-1β, and high mobility group box 1 [75]. The vascular leakage at this stage is largely functional, with key mechanisms including rapid cytoskeletal contraction induced by MLCK-mediated phosphorylation of MLC and enzymatic degradation of the eGC by enzymes such as heparinase and matrix metalloproteinase (MMP)-9 [123], [124]. These processes develop rapidly but can be reversed with timely interventions, such as pathogen removal and control of the inflammatory source.

In the late immunosuppressive phase of sepsis, continuous EC activation eventually leads to loss of cellular viability and large-scale programmed cell death. On the one hand, apoptosis is induced through death receptors such as Fas ligand and mitochondrial pathways, resulting in quiet and orderly death of ECs [125]; on the other hand, pyroptosis is mediated by inflammatory caspases such as caspase-1/-4/-5, cleaving Gasdermin D protein and forming pores in the cell membrane, resulting in lytic cell death and the release of endogenous danger signals such as IL-1β and IL-18, further exacerbating local inflammation and thrombosis [126], [127]. Critically, the endothelial repair mechanism is severely depleted. The Ang signaling axis becomes significantly imbalanced: pro-leak, unstable Ang-2 is continuously released, while the expression of protective and stable Ang-1 is significantly down-regulated [128]. The continuously increased Ang-2/Ang-1 ratio not only hinders vascular repair but also actively promotes inflammation and cell death [129]. Other repair pathways are also inhibited. For example, the function of vascular endothelial growth factor signaling shifts from promoting angiogenesis to exacerbating vascular leakage, and endogenous protective pathways such as S1P/S1PR1 signaling are also severely impaired [130]. S1P is a potent, well-validated endogenous regulator of endothelial barrier integrity, primarily due to the expression of membrane receptor S1PR1 on ECs [131], [132]. Its mechanism of action is primarily mediated through S1PR1, which, upon activation, initiates crucial cytoskeletal rearrangements. This includes the activation of small GTPases Ras-related C3 botulinum toxin substrate 1 (Rac1) and cell division control protein 42 homolog, which leads to the stabilization of AJs and the formation of a cortical actin ring [58]. These processes are fundamental for reducing vascular permeability and enhancing endothelial cohesion. These data support S1P/S1PR1 as a mechanistic barrier-protective axis in sepsis biology [57], [133]; however, clinical benefit remains unproven.

### 3.3 Mechanism of glycocalyx injury in sepsis

In sepsis, the eGC is among the earliest structures to be damaged, leading to endothelial dysfunction and increased vascular permeability [134], [135]. A key mechanism underlying this damage is the activation of neutrophils [136]. Once activated, neutrophils generate ROS via respiratory burst and release degradative enzymes, including myeloperoxidase, elastase, cathepsin G, and metalloproteinases, through degranulation, which together mediate the breakdown of glycocalyx components [137]. Concurrently, elevated levels of inflammatory mediators and chemokines promote further shedding of the glycocalyx, exposing adhesion molecules on the endothelial surface. This exposure allows selectins (E-, P-, and L-selectin) to initiate leukocyte rolling, followed by integrin-dependent firm adhesion via binding to ICAM-1 and vascular cell adhesion molecule-1 (VCAM-1), ultimately facilitating transendothelial migration of leukocytes [138]. These events not only amplify the inflammatory response but also activate the coagulation system, resulting in microcirculatory dysfunction and organ failure [139].

Glycocalyx components, such as hyaluronic acid, thrombomodulin, and SDC1 (CD138), enter the bloodstream and are soluble markers of endothelial activation and dysfunction [140], [141]. Glycocalyx damage enhances the interaction between neutrophils and ECs, further aggravating vascular leakage [142]. Studies have shown that IL-6 is closely associated with glycocalyx degradation in patients with coronavirus disease 2019 (COVID-19) or bacterial sepsis [143], [144], [145], [146]. In addition, sepsis increases the level of SDC1 in the eGC, while reducing the expression of TJ proteins ZO-1 and VE-cadherin, thereby promoting vascular leakage and increasing the risk of death [120].

In summary, endothelial barrier damage is central to the development of sepsis, leading to widespread vascular leakage, tissue edema, and organ hypoperfusion (Fig. 1**, left**); however, the mechanisms underlying this damage are highly heterogeneous. The diversity of these mechanisms suggests that achieving significant efficacy using a universal, single-target endothelial protection strategy is unlikely. Conversely, to effectively stabilize the endothelial barrier and prevent capillary leakage, individualized treatment based on precision medicine strategies is required.

## 4 Therapeutic strategies to protect endothelial barrier function in sepsis

As previously discussed, the mechanisms underlying endothelial dysfunction in sepsis are complex and heterogeneous, which makes the use of a single therapeutic approach challenging. Consequently, future treatment strategies should transition to a multi-target, comprehensive intervention model that can be dynamically adjusted based on the pathogen, host, and disease stage. This chapter will systematically review current treatment strategies for protecting the endothelial barrier, with a particular focus on glycocalyx preservation, junction stability (Fig. 1**, right**), and will explore the clinical prospects and challenges associated with these strategies (Table 1) [120], [147], [148], [149], [150], [151], [152], [153], [154], [155], [156], [157], [158], [159], [160], [161], [162], [163], [164], [165], [166], [167], [168], [169], [170], [171], [172], [173], [174], [175], [176], [177], [178], [179], [180], [181], [182], [183], [184], [185], [186], [187], [188], [189], [190], [191], [192], [193], [194], [195], [196], [197], [198], [199], [200], [201], [202], [203], [204], [205], [206], [207], [208], [209], [210], [211], [212], [213], [214], [215], [216], [217], [218], [219], [220], [221], [222], [223], [224], [225], [226], [227], [228], [229].

**Table 1:** Overview of therapeutic strategies for endothelial barrier protection in sepsis: mechanisms, evidence, and clinical applicability.

Strategy category	Representative agent/measure	Primary mechanism of action	Evidence level & Current status	Current clinical applicability	References
Inhibiting degrading enzymes	Low molecular weight heparin (LMWH)	Competitively inhibits heparanase	Preclinical efficacy; Clinically widely used for anticoagulation, endothelial protection is an ancillary benefit	Yes, for anticoagulation, not specifically for glycocalyx	[120], [147], [148], [149], [150], [151]
	Pixatimod (PG545)	Potent, anticoagulation-free heparanase inhibitor	Efficacy only in preclinical studies; In early-phase oncology clinical trials	No, not for sepsis indication	[152], [153], [154], [155], [156], [157]
Neutralizing inducing factors	N-acetylcysteine (NAC)	Antioxidant, reduces oxidative stress	Preclinical efficacy; Mixed results in small clinical RCTs	No, remains experimental/adjunctive	[158], [159], [160], [161], [162], [163], [164]
	Anti-TNF-α antibodies	Neutralizes the early key inflammatory cytokine	Preclinical efficacy; All large sepsis RCTs failed	No, no longer developed for sepsis	[165], [166], [167], [168]
Exogenous protection/replacement	Heparan sulfate analogs	Directly supplements the barrier, competitively inhibits enzymes	Efficacy only in preclinical studies	No	[169], [170], [171]
	Humanin derivatives (e.g., colivelin)	Pleiotropic: anti-inflammatory, antioxidant, anti-apoptotic, protects glycocalyx	Efficacy only in preclinical studies	No, it represents an emerging direction	[172], [173]
Modulating signaling pathways	Fingolimod	Activates S1P1 receptor, reinforces cell junctions and cytoskeleton	Strong preclinical evidence; Lacks large sepsis RCTs	No, used for multiple sclerosis	[174], [175], [176], [177], [178], [179], [180], [181], [182], [183], [184]
Optimized resuscitation strategy	Albumin	Maintains colloid osmotic pressure, may stabilize glycocalyx via binding	Large RCTs showed no mortality benefit	Yes, as a resuscitation fluid (non-specific)	[185], [186]
	Conservative crystalloid resuscitation	Avoids hemodilution and ANP-mediated glycocalyx shedding	Consensus based on physiology and observational studies	Yes, part of standard practice	[187], [188], [189], [190], [191], [192]
	Avoid hydroxyethyl starch (HES)	Avoids renal injury and direct endothelial damage	Numerous RCTs confirm harm	Contraindicated	[193], [194], [195], [196], [197]
Pleiotropic drugs	Statins	Anti-inflammatory, antioxidant, upregulates eNOS, potential glycocalyx protection	Observational studies suggest benefit; Sepsis RCTs are negative	Yes, for CV disease, continuing may be beneficial	[198], [199], [200], [201], [202], [203], [204], [205], [206], [207], [208], [209], [210]
	PCSK9 inhibitors (e.g., evolocumab)	Beyond lipid-lowering, it has anti-inflammatory properties, inhibits NF-κB, and reverses VE-cadherin internalization	Preclinical efficacy; Potential shown in small COVID-19 pilot trials	No (for hypercholesterolemia)	[211], [212], [213], [214], [215]
Targeting key pathways	Imatinib	Inhibits Abl kinase, enhances Rac1 activity, stabilizes junctions, anti-inflammatory	Preclinical efficacy; COVID-19 RCT showed reduced vascular leakage	No (for cancer, experimental for sepsis)	[216], [217], [218], [219]
	Selective V₁a receptor agonist (selepressin)	Avoids harmful effects of V_2_ receptor activation, reduces VEGF and Ang-2 expression	Good preclinical data; Large sepsis RCT did not show benefit	No	[225], [226]
Biomarker-guided therapy	Adrecizumab (anti-ADM antibody)	Partially neutralizes ADM, removing its harmful effects while preserving essential functions	Phase II trial showed improved SOFA score and a trend towards lower mortality	No, in clinical trials	[220], [221], [222], [223], [224]
Phenotype stratification	Activated protein C (APC)	Anticoagulant, anti-inflammatory, anti-apoptotic	Initial phase III success; Withdrawn after subsequent verification that the trial failed; Post-hoc analysis suggested efficacy in a hyperinflammatory phenotype	No (withdrawn), but a key exemplar of the precision medicine concept	[227], [228], [229]

TNF-α. Tumor necrosis factor-α; RCTs. Randomized controlled trials; S1P1. Sphingosine-1-phosphate receptor 1; ANP. Atrial natriuretic peptide; Enos. Endothelial NO synthase; CV. Cardiovascular; PCSK9. Proprotein convertase subtilisin/kexin type 9; NF-κB. Nuclear factor kappa B; VE-cadherin. Vascular endothelial cadherin; COVID-19. Coronavirus disease 2019; VEGF. Vascular endothelial growth factor; Ang-2. Angiopoietin-2; ADM. Adrenomedullin; SOFA. Sequential organ failure assessment

### 4.1 Protection and repair strategies of glycocalyx

The degradation of the glycocalyx in sepsis is a central mechanism of endothelial dysfunction. Therefore, protecting and repairing the glycocalyx has become a promising treatment direction. Current strategies primarily focus on the following aspects: inhibiting the activity of glycocalyx-degrading enzymes, blocking the factors that induce glycocalyx shedding, and providing exogenous substitutes or protective agents for the glycocalyx. These approaches aim to preserve glycocalyx integrity and function, thereby improving endothelial barrier stability and clinical outcomes.

#### 4.1.1 Inhibition of glycocalyx-degrading enzymes

*Heparanase inhibitor* Heparanase is a key enzyme that directly hydrolyzes the side chain of HS. Inhibiting its activity can directly reduce the shedding of HS. In addition to its anti-coagulation, low molecular weight heparin (LMWH) is a competitive inhibitor of heparanase. Preclinical studies using rat cecal ligation and puncture model have shown that LMWH can reduce SDC1 shedding, vascular leakage, and organ damage [147], [148]. Some small-scale clinical observational studies suggest that plasma SDC1 levels are lower in patients with sepsis who use prophylactic anti-coagulation [120], [149], [150], [151]. However, as glycocalyx protection is not a primary endpoint in large randomized controlled trials (RCTs), and its benefits may be conflated with or confounded by its anticoagulant effects, there is insufficient evidence to recommend its use specifically for glycocalyx protection at this time.

Novel heparinase inhibitors, such as pixatimod (PG545), are designed to provide potent heparinase inhibition without anticoagulant activity [152]. A large number of preclinical studies (in vitro cell model, mouse models of various diseases) have shown their effectiveness in stabilizing the glycocalyx, inhibiting tumor metastasis, and reducing inflammation [153], [154], [155], [156], [157]. However, these inhibitors are currently in the early clinical research stage for cancer treatment and have not yet started clinical trials for sepsis.

*Inhibitors of MMP* In sepsis, MMPs exacerbate glycocalyx shedding and organ damage by cleaving the protein core of the glycocalyx and activating inflammatory factors. Broad-spectrum MMP inhibitor doxycycline is a tetracycline antibiotic. In addition to antibacterial activity, it also has the ability to non-specifically inhibit MMP (especially MMP-9). Preclinical studies in sepsis mouse models have shown that it reduces glycocalyx damage and organ dysfunction [230], [231], [232]. Although few small clinical studies have explored its use in patients with sepsis or acute respiratory distress syndrome (ARDS), most of them focused on its antibacterial effect or mild immunomodulatory effect, with limited high-quality evidence regarding its MMP inhibition and impact on clinical outcomes [233], [234]. At present, no specific MMP inhibitors have been approved for the treatment of sepsis, and this area remains primarily in the basic research phase.

#### 4.1.2 Blocking the cause of calyx shedding

*Antioxidant therapy* N-acetylcysteine (NAC), an antioxidant, has been shown to reduce glycocalyx damage in preclinical studies [161], [162]. Several small RCTs involving patients with sepsis or ARDS have studied the effect of NAC, but the results are inconsistent [158], [159], [160]. While some trials indicate that NAC can improve biomarkers of oxidative stress, consistent beneficial effects on key clinical outcomes, including glycocalyx integrity-related indicators (such as SDC1) or mortality, have not been demonstrated [163], [164]. Therefore, it is still regarded as an experimental adjuvant therapy.

Vitamin C has also been investigated for the treatment of sepsis. In sepsis mouse models, vitamin C reduced not only the dephosphorylation of occludin, but also decreased the excessive production of NO and ROS, as well as vascular leakage [235]. Despite much good preclinical evidence, several large multicenter RCTs unfortunately failed to demonstrate consistent improvements in key clinical outcomes [236]. Thus, its protective effect on glycocalyx is primarily based on preclinical evidence, which has not been substantiated in large clinical trials.

*Anti-inflammatory treatment* Blocking early inflammatory factors can inhibit the activation of its downstream signaling pathways, thereby preventing glycocalyx shedding. For example, anti-TNF-α is effective in many preclinical models of sepsis [165]. However, it failed in large clinical RCTs and did not improve the prognosis of patients with sepsis [166], [167], [168]. This suggests that in complex clinical settings, blocking a single early inflammatory factor may be too late or insufficient to change the course of the disease. Therefore, a combination therapy strategy warrants further exploration in clinical studies.

#### 4.1.3 Exogenous glycocalyx protection and substitution agents

HS analogs (especially non-anticoagulant heparin and heparin itself) are theoretically an ideal strategy for protecting the glycocalyx in sepsis by replacing degraded HS, neutralizing inflammatory mediators, and inhibiting degrading enzymes [120]. Even though sufficient preclinical evidence has been gathered, translating these findings into effective clinical treatment remains challenging due to the risk of bleeding and the complexity of the disease [169], [170], [171].

Humanin, a short 21-24 amino acid mitochondrial peptide, and its derivatives (e.g., humanin, colivelin) are endogenous peptides that have strong cyto-protective, anti-apoptotic, and anti-inflammatory properties [172]. In cellular and animal models, humanin analog colivelin directly antagonizes factors that induce glycocalyx injury such as inflammation, oxidative stress, and effectively protects the endothelial barrier [173]. While these preclinical evidences support the encouraging protective potential of these peptides, their clinical applicability remains to be established through further translational studies.

### 4.2 Targeting endothelial signaling pathways: S1P and its receptor agonists

Fingolimod (FTY720), a S1P receptor modulator, has been approved for the treatment of multiple sclerosis in the clinic [174]. Notably, it is also an agonist of the endothelial barrier [175]. Some preclinical studies (animal models of sepsis [176] and acute lung injury [177]) have shown that fingolimod significantly reduces vascular leakage, inflammation, and tissue edema [178], [179], [180], [181], [182]. Preliminary evidence also suggests potential benefits in the treatment of ARDS or COVID-19-associated acute respiratory failure [183], [184]. However, there is currently a lack of large-scale RCTs to support its use in sepsis, and its barrier protection may be compromised by its immunosuppressive properties in clinical applications.

Other selective S1P receptor agonists (e.g., Ceralifimod, Ozanimod) are a new generation of drugs designed to enhance receptor selectivity. At present, they are primarily in the clinical trial phase of autoimmune diseases [237], [238]. This approach represents a novel therapeutic strategy, distinct from traditional anti-inflammatory paradigms.

### 4.3 Colloid resuscitation and hemodynamic optimization

#### 4.3.1 Albumin

Albumin, a natural colloid with superior intravascular persistence compared to crystalloids, is believed to be beneficial due to its role in stabilizing the glycocalyx (as an integral component) and its potent antioxidant effects [185]. The large multicenter albumin Italian outcome sepsis RCT found no significant difference in 28-day mortality between albumin and crystalloid resuscitation in sepsis, although potential trends were noted in certain subgroups [186]. This also suggests that its glycocalyx protective effect is more inferential, rather than directly confirmed by clinical trials.

#### 4.3.2 Avoiding excessive hemodilution represents a key strategy for glycocalyx protection

The physiological rationale is that over-infusion of crystalloids dilutes plasma protein concentration, thereby disrupting the equilibrium of intravascular Starling forces. This imbalance promotes fluid leakage into the interstitial space, exacerbating tissue edema [187]. More importantly, volume expansion stimulates the release of atrial natriuretic peptide, which is a well-established mediator that potentiates glycocalyx shedding [188], [189]. Consequently, this approach is supported by physiological principles and observational studies [190], [191], [192], highlighting that precise and conservative liquid management serves as a glycocalyx protection strategy.

Synthetic colloids, such as hydroxyethyl starch (HES), have been shown in numerous high-quality clinical RCTs and meta-analyses to increase the risk of acute kidney injury and mortality in patients with sepsis [193], [194], [195]. Therefore, given that HES is contraindicated in sepsis resuscitation, it should not be considered a viable option for glycocalyx-protective therapy [196], [197]. This limitation underscores the urgent need to identify and validate effective alternatives. Despite a robust pathophysiological rationale and promising preclinical data, the vast majority of specific glycocalyx-protective strategies have yet to be translated into clinically proven therapies. Currently, the most direct clinical implications involve avoiding the use of known harmful fluids (e.g., HES) and employing meticulous fluid management to prevent iatrogenic glycocalyx injury. Future translational research needs to focus on discovering biomarkers (such as SDC1) that enable rapid assessment of glycocalyx integrity at the bedside, which will facilitate the development of precision clinical trials with targeted interventions for specific patient populations.

### 4.4 Multi-target integrated stabilization strategy

The pathological mechanism of endothelial injury in sepsis is highly complex, involving multiple parallel and interconnected processes such as inflammatory cascades, oxidative stress, glycocalyx shedding, and disruption of intercellular junctions. This multifaceted nature renders that single-target intervention strategies often prove ineffective. Consequently, research perspectives have gradually shifted toward agents with “pleiotropic effects”: compounds capable of synergistically stabilizing endothelial function through multiple molecular pathways. The core rationale of this approach lies in simultaneously modulating multiple key nodes to reestablish homeostasis within the internal environment, rather than merely blocking an isolated pathway.

#### 4.4.1 Statins

Statins are a typical representative of this strategy. Extensive preclinical studies have confirmed that statins (such as simvastatin) not only exert anti-inflammatory and antioxidant effects by upregulating eNOS and inhibiting the nuclear translocation of NF-κB [198], [199], [200], [201], [202], [203], but also mitigate LPS-induced endothelial hyperpermeability by enhancing the activity of cytoskeletal regulatory factors such as IQ motif containing GTPase activating protein 1 [182], [203], [204], [205], [206]. Accumulating evidence from observational studies suggests that prior statin use is associated with improved survival in sepsis patients [207], [208], [209]; however, well-designed RCTs have failed to replicate a significant mortality benefit [210]. This paradox suggests that the mechanistic efficacy of a drug does not necessarily translate into broad clinical effectiveness, potentially due to unrecognized patient heterogeneity hidden beneath the surface, laying the groundwork for subsequent “precision medicine” considerations.

#### 4.4.2 Proprotein convertase subtilisin/kexin type 9 (PCSK9) inhibitors

PCSK-9 inhibitors, as emerging pleiotropic agents, offer value beyond lipid-lowering effects [211], [212]. In the context of sepsis, PCSK9 levels are significantly elevated, where it promotes endothelial dysfunction through TLR4/NF-κB and NOD-, LRR- and pyrin domain-containing protein 3 inflammasome pathways [213]. Studies have shown that PCSK9 is upregulated during sepsis and directly participates in exacerbating inflammatory responses and disrupting endothelial junctions (e.g., by reducing VE-cadherin expression) [213], [214]. Inhibition of PCSK9 has shown potential in preclinical models to alleviate organ damage and inflammation. More notably, a pilot trial in critically ill COVID-19 patients found that treatment with evolocumab significantly reduced IL-6 levels, decreased the need for invasive ventilation, and improved mortality rates [215], underscoring its considerable potential as an endothelial barrier-protective agent.

#### 4.4.3 Imatinib

Imatinib stabilizes the endothelial barrier by inhibiting Abl family tyrosine kinases, thereby preventing the disruption of AJs and the formation of intercellular gaps [216]. Simultaneously, it enhances the activity of Rac1, a barrier-supportive GTPase, synergistically reinforcing the stability of the VE-cadherin complex [217]. Additionally, imatinib exhibits immunomodulatory effects and has been shown to attenuate inflammation in LPS-induced animal models of lung injury [218]. This multifaceted mechanism, concurrently targeting barrier integrity, cytoskeletal dynamics, and the immune system, makes it a highly attractive candidate for repurposing. Its potential has been preliminarily validated in an RCT involving critically ill COVID-19 patients, offering promise for its application in sepsis, although no sepsis-specific RCT evidence to date [219].

#### 4.4.4 Adrenomedullin (ADM)

ADM is a potent vasoactive peptide hormone, and its plasma concentration increases sharply in sepsis, and the levels are directly correlated with the severity of vascular paralysis and mortality [221]. ADM has been shown to enhance EC junction integrity and reduce vascular permeability in many preclinical experiments [220]. In a staphylococcal toxin-induced sepsis rat model, exogenous ADM supplementation significantly reduced the mortality rate from 53% to 7%, underscoring its substantial therapeutic potential [222]. Adrecizumab is a humanized monoclonal antibody directed against ADM. In LPS and cecal ligation and puncture rat models, adrecizumab exhibits significant endothelial protection [223]. In the subsequent adrenomedullin and outcome in severe sepsis and septic shock 2 study, a faster improvement in organ function (sequential organ failure assessment score) and a trend toward reduced 28-day mortality (23.9% vs 27.7%) were observed in patients with high proADM septic shock treated with adrecizumab, further confirming its clinical feasibility [224].

### 4.5 Historical failures offer compelling counterevidence for precision medicine

Despite the mechanistic promise of the aforementioned strategies, the history of sepsis treatment is filled with failures in pivotal clinical trials. These failures primarily result from the substantial pathophysiological heterogeneity of sepsis and the diversity of patient phenotypes [239]. This imperative compels a shift in our therapeutic approach from a broad-spectrum treatment model towards a precision medicine paradigm.

#### 4.5.1 Activated protein C (APC)

The trajectory of APC is marked by notable shifts in its clinical and research status. It was initially approved based on an RCT demonstrating a reduction in the absolute mortality rate, only to be withdrawn from the market after subsequent confirmatory trials failed to replicate the benefit and indicated an associated risk of bleeding [227]. The key to this reversal lies in post-hoc analyses, which revealed that APC exerted opposed effects depending on the inflammatory phenotype of the patients. It significantly reduced mortality in those with a hyper-inflammatory phenotype, while potentially causing harm in patients with a hypo-inflammatory phenotype [228], [229]. This case powerfully demonstrates that without phenotypic stratification, clinical trials may not only fail to identify effective treatments but also conceal significant benefits, or even harms, for specific patient subgroups.

#### 4.5.2 Selepressin

Selepressin, a selective V_1_a receptor agonist, has been shown to significantly reduce vascular leakage in animal models of sepsis by specifically activating of V_1_a receptor, thereby increasing vascular resistance while avoiding adverse effects linked to V_2_ receptor activation such as vasodilation, endothelial activation, and increased vascular permeability, thereby effectively reducing fluid extravasation and tissue edema [225]. However, this promising preclinical efficacy did not translate into improved patient outcomes in subsequent large human RCTs [226]. This further underscores that animal models cannot fully simulate the heterogeneity of human diseases.

## 5 Conclusions

Endothelial dysfunction represents a dynamic and central component of sepsis pathophysiology, influenced by variable factors such as pathogen characteristics, host age, and comorbidities. This multifactorial injury network limits the efficacy of single-target therapies, driving the development of multi-target and precision-based treatment approaches. Emerging consensus suggests that therapeutic breakthroughs will require integrated strategies beyond conventional interventions. These include the developing pleiotropic agents capable of stabilizing endothelium, modulating inflammation, and regulating immune activity, alongside biomarker-guided phenotyping for precise patient stratification. By dissecting the heterogeneity of sepsis, tailored interventions can be aligned with specific endothelial injury endotypes, enabling mechanism-driven, individualized therapies. Looking ahead, the management of endothelial dysfunction in sepsis is poised to shift from empirical care toward precision medicine. This transition will depend on integrating mechanistic insights from research with dynamic and rapid phenotyping in clinical practice, ultimately advancing an endotype-based personalized treatment paradigm with the potential to improve outcomes in septic patients.

## Abbreviations

Ang: Angiopoietin

AJs: Adherens junctions

APC: Activated Protein C

ARDS: Acute respiratory distress syndrome

ADM: Adrenomedullin

ACE2: Angiotensin-converting enzyme 2

COVID-19: Coronavirus disease 2019

EC: Endothelial cell

eGC: Endothelial glycocalyx

eNOS: Endothelial nitric oxide synthase

HES: Hydroxyethyl starch

HS: Heparan sulfate

ICAM-1: Intercellular adhesion molecule-1

IEJs: Interendothelial junctions

IL: Interleukin

LPS: Lipopolysaccharide

LMWH: Low molecular weight heparin

MAPK: Mitogen-activated protein kinase

MLCK: Myosin light-chain kinase

MLC: Myosin light chain

MMP: Matrix metalloproteinase

NO: Nitric oxide

NF-κB: Nuclear factor kappa B

NOS: Nitric oxide synthase

NAC: N-acetylcysteine

PAI-1: Plasminogen activator inhibitor-1

PAR: Protease-activated receptor

PCSK9: Proprotein convertase subtilisin/kexin type 9

PG545: Pixatimod

Rac1: Ras-related C3 botulinum toxin substrate 1

RCTs: Randomized controlled trials

RIG-I: Retinoic acid-inducible gene I

RhoA: Ras homolog gene family member A

ROS: Reactive oxygen species

SARS-CoV-2: Severe acute respiratory syndrome coronavirus 2

SDC1: Syndecan-1

S1P: Sphingosine-1-phosphate

TLR: Toll-like receptor

TNF-α: Tumor necrosis factor-α

TJs: Tight junctions

TGF-β: Transforming growth factor-β

TF: Tissue factor

TFPI: Tissue factor pathway inhibitor

vWF: Von Willebrand factor

VE-cadherin: Vascular endothelial cadherin

ZO: Zona occludens

## Ethics approval and consent to participate

Not applicable.

## Authors’ contributions

YHJ, XJL, and RFM wrote the manuscript. YHJ and RFM drew the figures. YL, DCM, YYS, and YLC helped modify the figures. YHJ, RFM, and YL revised the manuscript. RFM and YL conceived and supervised the study. All authors read and approved the final manuscript.

## Funding

This work was supported by the National Natural Science Foundation of China (82570582, 82070505), the Distinguished Professorship Program of Jiangsu Province to Ren-Fang Mao, the Nantong University Clinical Medicine Special Research Fund (2024JZ036), and the Postgraduate Research & Practice Innovation Program of Jiangsu Province (KYCX25_3788).

## Competing interests

The authors declare that they have no competing interests.

## Data Availability

Not applicable.
